# Linking bisphenol a exposure to MASLD: insights from network toxicology and machine learning based on NHANES 2005–2012 data

**DOI:** 10.1093/toxres/tfaf165

**Published:** 2025-11-25

**Authors:** Jiaquan Yuan, Haoyang Xu, Junhong Gan, Haiyan Zhao

**Affiliations:** Graduate School of Guangxi University of Chinese Medicine, Nanning, Guangxi 530020, China; Graduate School of Guangxi University of Chinese Medicine, Nanning, Guangxi 530020, China; Graduate School of Guangxi University of Chinese Medicine, Nanning, Guangxi 530020, China; Yongning District Hospital of Traditional Chinese Medicine, Nanning, Guangxi 530200, China

**Keywords:** network toxicology, machine learning, immune cell infiltration analysis, molecular dynamics simulation, bisphenol a, metabolic steatotic liver

## Abstract

Metabolic Dysfunction-Associated Steatotic Liver Disease (MASLD), one of the most prevalent chronic liver diseases worldwide, has a pathogenesis that remains incompletely understood. In recent years, Bisphenol A (BPA) has been recognized as an emerging pathogenic factor for MASLD as an environmental contaminant. By integrating multiple advanced methodologies including Network Toxicology, Machine Learning, Molecular Docking, and Molecular Dynamics Simulation, this study systematically elucidates the molecular mechanisms underlying BPA-induced MASLD. Through analysis of NHANES data, we identified a significantly positive correlation between BPA levels and MASLD risk. Integration of multiple databases identified 34 potential BPA-related targets. KEGG enrichment analysis revealed the critical role of the PI3K/AKT signaling pathway in MASLD, with COL1A1, COL1A2, and IGF1 serving as core targets that drive disease progression. Immune cell infiltration analysis demonstrated that BPA regulates immune cell function via the PI3K/AKT pathway, thereby promoting the onset and development of MASLD. These findings reveal the complex mechanisms underlying BPA-induced MASLD and provide novel therapeutic targets, along with theoretical support for the early screening and precision treatment of this disease.

## Graphical Abstract

**Graphical Abstract:**
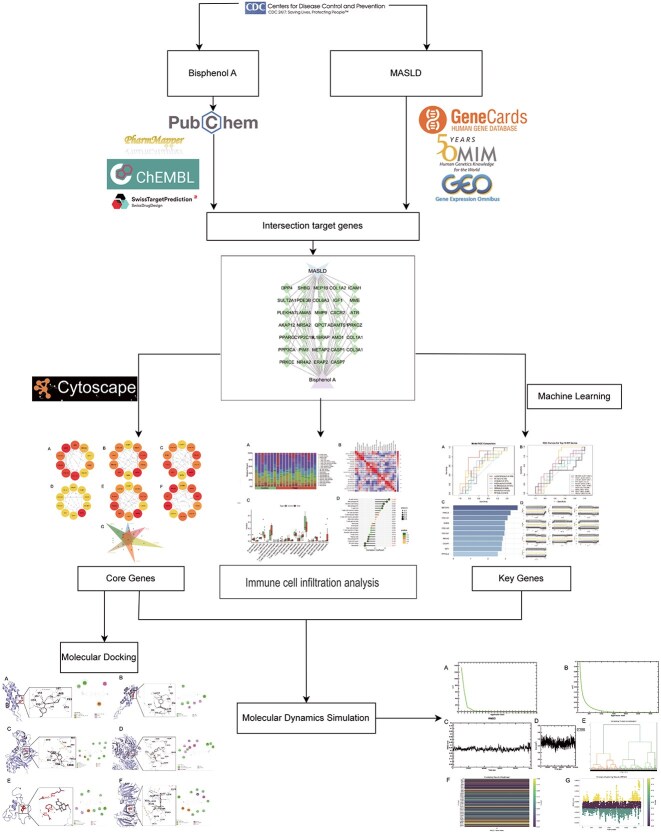

## Introduction

In recent years, as the understanding of the impact of environmental chemical exposure on metabolic diseases has deepened, endocrine disruptors such as Bisphenol A (BPA) have been recognized as emerging pathogenic factors for Metabolic Dysfunction-Associated Steatotic Liver Disease (MASLD).^1^ MASLD proposed in 2020, replacing the previous `Non-Alcoholic Fatty Liver Disease (NAFLD)’. This standardization of nomenclature signifies both a deepened understanding of the disease and optimized classification, emphasizing its intrinsic link with metabolic abnormalities.^2^^,^^3^ MASLD currently affects approximately 30% of the global adult population, making it one of the most prevalent chronic liver diseases worldwide, and its incidence continues to increase.^4^ Although MASLD often presents without overt clinical symptoms in its early stages, the condition can progress to metabolic dysfunction-associated steatohepatitis (MASH), subsequently leading to hepatic fibrosis, liver cirrhosis, and ultimately hepatocellular carcinoma (HCC). Research indicates that The pathogenesis of this disease is closely associated with multiple metabolic dysregulation mechanisms, including collaborative interactions across pathways, such as insulin resistance, oxidative stress, and carbonyl stress,^3^^,^^5^ Furthermore, MASLD can induce cardiovascular diseases, with MASH patients exhibiting an annual mortality rate of approximately 2.6%, primarily attributed to cardiovascular complications and HCC as leading causes of death^6^ Major risk factors for MASLD include obesity, type 2 diabetes mellitus, insulin resistance, and dyslipidemia. These metabolic disorders not only promote hepatic fat deposition but also significantly increase the risk of progression to MASH and hepatic fibrosis^7–9^ Consequently, MASLD is not merely a liver disease but also a systemic metabolic disorder.

Bisphenol A (BPA) is a water-soluble compound classified as a diphenylmethane derivative. This chemical is ubiquitously present in most polycarbonate plastics and epoxy resins.^10^ It is released upon heating and is recognized for its endocrine-disrupting effects that mimic estrogen.^11^ Primarily used in plastics, food packaging, and other consumer products, it poses hazards to human health.^12^ Research indicates that dietary exposure, primarily through BPA-contaminated food and beverages, constitutes the principal route of human BPA exposure.^13^^,^^14^ Under normal use conditions, BPA may migrate from polycarbonate materials into food or drinking water. The migration of BPA involves both physical diffusion and chemical degradation of the polymer during use, particularly hydrolysis reactions. BPA detected in food may originate from unreacted residual monomers in the material or be released by the decomposition of polycarbonate under specific environmental conditions.^15^ BPA is carcinogenic, and serum BPA levels show significant association with prostate cancer risk.^16^ BPA can also induce neurotoxicity,^17^ leading to metabolic disorders (obesity, diabetes mellitus) and cardiovascular diseases.^18^

BPA is closely associated with MASLD. Extensive animal and cellular experiments demonstrate that BPA promotes hepatic lipid droplet accumulation and accelerates MASLD onset and progression by disrupting lipid metabolism, inducing insulin resistance, triggering oxidative stress, and interfering with hepatocellular mitochondrial function.^19^^,^^20^ Low-dose BPA exposure can downregulate DNA methylation levels in the promoter region of hepatic SREBP-2, enhancing its transcriptional activity and promoting the expression of genes involved in cholesterol synthesis. This leads to intrahepatic cholesterol accumulation, further activates lipid synthesis pathways, and ultimately triggers hepatic steatosis.^21^ Concurrently, population-based epidemiological studies support this perspective. Multiple cross-sectional investigations have revealed significant correlations between urinary BPA levels and elevated transaminases (ALT/GGT) as well as MASLD diagnostic indicators, with particularly pronounced associations observed in children and adolescents.^22^ However, systematic mechanistic research on BPA-induced MASLD remains fragmented, lacking integrated molecular mechanisms and sufficient elucidation of the key molecular targets driving MASLD pathogenesis. To decipher its complex toxic pathways, this study systematically analyzed the potential molecular mechanisms and core genes by employing multiple methodologies, including Network Toxicology, Machine Learning, molecular docking, and molecular dynamics simulation, thereby providing a scientific basis for the early screening and precision treatment of MASLD associated with Bisphenol A exposure.

## Methods

### Analytical methods based on NHANES data

This study utilized data from the National Health and Nutrition Examination Survey (NHANES) spanning 2005–2012 to investigate the association between urinary Bisphenol A (BPA) levels and the risk of nonalcoholic fatty liver disease (MAFLD). The NHANES, conducted by the Centers for Disease Control and Prevention (CDC), encompasses nationally representative samples across age groups, ethnicities, and geographic regions. This multi-year epidemiological survey has been extensively utilized in public health and epidemiological research. The dataset employed in this study incorporated participants' health examination records, dietary questionnaires, and biological specimens, providing comprehensive information on physiological status, metabolic profiles, and environmental exposures. The data underwent standardized processing, including missing value imputation and outlier detection, to ensure the accuracy and reliability of the research outcomes. BPA exposure levels were categorized into four quartiles (BPA_Q1 to BPA_Q4) and analyzed as primary independent variables. Risk assessment for MAFLD utilizes two common scoring methods: the Hepatic Steatosis Index (HSI) and the Ultrasound-based Fatty Liver Index (USFLI). Specifically, HSI was calculated using the following formula: HSI = 8 × (ALT/AST) + BMI + 2 × sex (1 for male, 2 for female) + 2 × Diabetes Mellitus history. Participants with HSI > 36 were classified into the high-risk group. To further analyze the relationship between BPA exposure and MAFLD risk, we employed a multivariate logistic regression model. The model incorporated variables, including BPA quartiles, BMI, age, sex, and history of diabetes mellitus. Statistical methods, such as the area under the receiver operating characteristic curve (AUC), chi-square test, and trend analysis, were employed to ensure model stability and accuracy. Multicollinearity analysis was also integrated into the model validation to eliminate potential confounding effects.

### Acquisition of Bisphenol a targets

The molecular structure of Bisphenol A was obtained from the PubChem database (https://pubchem.ncbi.nlm.nih.gov/). Potential targets were identified using SwissTargetPrediction (http://www.swisstargetprediction.ch/, PharmMapper (http://www.lilab-ecust.cn/pharmmapper/), ChEMBL (https://www.ebi.ac.uk/chembl/). Target names were converted to gene names using UniProt (https://www.uniprot.org/).

### Acquisition of MASLD targets

Using GeneCards (https://www.genecards.org/) and OMIM (https://www.omim.org/) with `Metabolic Associated Fatty Liver Disease' or `Non-Alcoholic Fatty Liver Disease' as keywords, targets obtained from all databases were merged, and duplicate values were eliminated to acquire disease genes.

### Acquisition and Preprocessing of GEO datasets

This study employed three microarray datasets (GSE48452, GSE63067, and GSE66676) from the GEO platform (https://www.ncbi.nlm.nih.gov/gds) to screen differentially expressed genes and conduct batch effect analysis. Raw data files were downloaded from the GEO database and preprocessed using R programming language. The preprocessing steps included the removal of low-expression genes, standardization, and batch effect correction. Batch effect removal was performed using the ComBat algorithm, which adjusts for systematic differences between batches. Following data processing, we employed Principal Component Analysis (PCA) to conduct a dimensionality reduction analysis on the processed data. This facilitated the examination of sample distributions across different datasets and the assessment of batch effects. PCA was performed using the prcomp() function, extracting the first two principal components (PC1 and PC2). Subsequently, significantly differentiated genes (Differentially Expressed Genes, DEGs) were screened based on the criteria of |logFC| > 0.5 and *P*-value <0.05. All data analyses and visualization tasks were completed using R software packages.

### Machine learning analysis

To further explore the key genes associated with Bisphenol A (BPA), we applied multiple machine learning models for feature selection and predictive analysis. Initially, classical machine learning models, including Random Forest (RF), Support Vector Machine (SVM), and Logistic Regression (LR), were employed for preliminary training and feature evaluation. The performance of each model was assessed using cross-validation and the Area Under the Curve (AUC), with the results visualized using Receiver Operating Characteristic (ROC) curves. To evaluate the importance of each gene in the model predictions, we applied the Shapley additive explanation (SHAP) method. SHAP values elucidate the contribution and influence of features in model predictions, thereby facilitating the interpretation of the model's decision-making process. The training process and performance evaluation of all models were based on 34 overlapping genes screened from BPA targets and disease-related genes.

Based on the AUC values of each model, we selected the best-performing Random Forest (RF) model. The RF model demonstrates significant advantages in handling high-dimensional data and automatically processing missing values while being less prone to overfitting. These characteristics endow it with high stability and accuracy when processing complex genetic data sets. Using the RF model, we screened the top 10 genes with the highest AUC values and further evaluated their importance through AUC and SHAP analyses. Ultimately, three genes with AUC values greater than 0.6 were selected as key biomarkers associated with BPA exposure.

### Construction of PPI network and screening of core targets

Disease-associated genes obtained from the GeneCards and OMIM databases were intersected with the significantly differentially expressed genes screened from the GEO database. The resulting overlapping genes were designated as the total disease-gene set. Subsequently, these disease genes were further intersected with the target genes of BPA to identify potential targets for MASLD. The intersection results were visualized using Jvenn (https://jvenn.toulouse.inra.fr/app/example.html).

The identified targets were imported into the STRING database, with the species limited to *Homo sapiens* and a medium confidence score threshold (>0.4) applied for analysis. The interaction network generated by STRING was imported into Cytoscape 3.10.2 to construct a protein–protein interaction (PPI) network.

For a comprehensive analysis, the CytoHubba plugin was employed to calculate the top 10 hub genes using multiple algorithms, including Maximum Clique Centrality (MCC), Maximum Neighborhood Component (MNC), Degree, EcCentricity, Radiality, and Stress. The intersection network derived from these algorithms was analyzed to identify the core targets for subsequent investigation.

### GO and KEGG pathway analysis

Gene Ontology (GO) functional annotation and Kyoto Encyclopedia of Genes and Genomes (KEGG) pathway enrichment analysis were conducted using the “ClusterProfiler,” “Enrichplot,” and “Org.Hs.eg.db” packages in R software to investigate potential genes associated with bisphenol A-induced fatty liver disease (MASLD). The analysis covered biological processes (BP), molecular functions (MF), and cellular components (CC). Key pathways related to potential targets of bisphenol A-induced MASLD were identified and visualized through KEGG enrichment analysis.

### Molecular docking

Molecular docking analysis was performed to examine the binding modes and affinity between Bisphenol A core targets. The 3D structure of Bisphenol A was retrieved from the PubChem database (https://pubchem.ncbi.nlm.nih.gov/), while the 3D structures of core genes were obtained from the Protein Data Bank (PDB; https://www.rcsb.org/). AutoDock Vina was employed for docking, followed by validation using PyMOL and CB-Dock 2 (https://cadd.labshare.cn/cb-dock2/php/index.php). Subsequently, the Discovery Studio 2025 Client was used to generate 2D binding diagrams.

### Molecular dynamics simulation

This study utilized GROMACS version 2025.1 to conduct molecular dynamics simulations. The ligand topology file was generated using the CHARMM36 force field (including the TIP3P water model), with energy minimization performed via the steepest descent and conjugate gradient methods. Following energy minimization, we conducted a 100 ns NVT equilibration simulation, gradually heating the system to 300 K. Subsequently, the system underwent re-equilibration under isothermal-isobaric (NPT) conditions. Finally, a 200 ns molecular dynamics production simulation was performed.

Throughout the simulation, in addition to conventional analytical methods such as RMSD and SASA, we performed PCA and cluster analyses to evaluate system stability and characteristics across different states.

### Cellular infiltration analysis

To preliminarily evaluate the immune system’s response during disease progression, the relative abundance of 22 immune cell types in each sample was estimated based on the merged gene expression matrix using the CIBERSORT algorithm. CIBERSORT applies a linear support vector regression model to deconvolute transcriptomic data, thereby inferring the proportions of diverse immune cell populations. A stacked bar chart was generated to intuitively display the differences in immune cell composition, while a correlation heatmap illustrated potential synergistic or antagonistic relationships among immune cell subsets. Boxplots were further employed to compare the infiltration levels of various immune cell types between groups. Finally, Spearman correlation analysis was conducted to determine the associations between core genes and immune cell subsets, providing supporting evidence for subsequent mechanistic discussions.

## Results

### Association analysis between BPA exposure and MAFLD risk

We analyzed the relationship between urinary BPA levels and MAFLD risk in 35,823 participants from the NHANES 2005–2012 period. MAFLD prevalence was assessed using both HSI and USFLI scores. The findings indicate a significant positive correlation between BPA exposure levels and MAFLD risk.

#### Association between BPA exposure and MAFLD risk

We observed a significant trend between BPA levels and MAFLD risk. The logistic regression model results revealed that compared with the lowest quartile of urinary BPA (BPA_Q1), individuals in the third (BPA_Q3) and fourth (BPA_Q4) quartiles exhibited significantly increased odds of high MAFLD risk (BPA_Q3: OR = 1.69, 95% CI 1.39–2.04; BPA_Q4: OR = 1.44, 95% CI 1.19–1.76; *P* < 0.001). This indicates a positive correlation between higher BPA levels and increased MAFLD risk, as shown in Table 1.

**Table 1:** Multivariate logistic regression analysis results (MAFLD risk prediction).

Variable	Estimate	Standard error	Z-value	P-value
(Intercept)	−3.733	0.102	−36.606	< 2e-16
BPA Quartile Q2	−16.76	115	−0.146	0.884
BPA Quartile Q3	0.221	0.061	3.618	0.0003
BPA Quartile Q4	1.204	0.053	22.905	< 2e-16
BMXBMI	0.0217	0.0027	7.711	1.25e-14
Age (RIDAGEYR)	0.0125	0.0009	13.475	< 2e-16
Gender (RIAGENDR)	−0.0994	0.041	−2.404	0.0162
Diabetes (HAS_DIABETES)	0.1429	0.064	2.255	0.0241

After adjusting for potential confounders, including BMI, age, sex, and diabetes mellitus, the multivariate logistic regression model further confirmed an independent association between BPA levels and MAFLD risk. Specifically, the fourth quartile of BPA exposure exhibited an OR of 1.44 (95% CI 1.05–1.98; *P* = 0.012), demonstrating an independent relationship between BPA levels and MAFLD risk. These results remained significant after adjusting for other covariates (such as Body Mass Index and Diabetes Mellitus status). As shown in Table 2.

**Table 2:** Trend analysis results—Relationship between BPA quartiles and MAFLD risk.

Variable	Estimate	Standard error	Z-value	P-value
(Intercept)	−5.217	0.112	−46.666	< 2e-16
BPA Quartile Trend (BPA_Q_trend)	0.6288	0.0203	30.919	< 2e-16
BMXBMI	0.0229	0.0028	8.267	< 2e-16
Age (RIDAGEYR)	0.0129	0.0009	13.628	< 2e-16
Gender (RIAGENDR)	−0.1045	0.041	−2.562	0.0104
Diabetes (HAS_DIABETES)	0.1303	0.065	2.013	0.0441

#### Model performance

The performance of the Logistic Regression Model was evaluated using AUC, yielding a value of 0.76. This indicates strong discriminatory and predictive capabilities, enabling the effective identification of individuals at high risk of MAFLD.

#### Trend analysis

Trend analysis of BPA exposure levels revealed a consistent increase in MAFLD risk with increasing BPA quartiles. After converting the BPA quartiles (Q1 to Q4) into a numerical variable for trend testing, the results demonstrated a significant positive correlation between increasing BPA levels and elevated MAFLD risk (OR per unit increase in BPA_Q_trend = 1.63, 95% CI 1.53–1.74, *P* < 0.001). This indicates a dose-dependent relationship between BPA exposure and MAFLD development. As presented in Table 3.

**Table 3:** Stacked bar chart analysis of BPA quartiles and MAFLD risk labels.

BPA quartile	High risk	Intermediate risk	Low risk
Q1	544	9,654	0
Q2	0	10,198	0
Q3	623	9,574	0
Q4	1,593	8,604	0

These findings collectively demonstrate a strong association between BPA exposure and MAFLD risk, highlighting the need for further research on the mechanisms through which BPA may contribute to liver disease pathogenesis.

### Integration of GEO datasets and differential expression analysis results

**Fig. 1:**
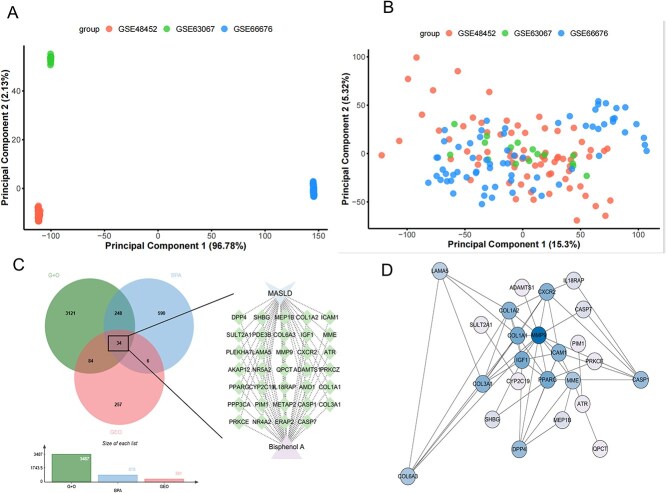
A) PCA plot before batch effect correction, showing the distribution of samples from the three GEO datasets: GSE48452, GSE63067, and GSE66676. PC1 explains 96.78% of the variance, and PC2 explains 2.13% B) PCA plot after batch effect correction, with PC1 explaining 15.3% of the variance and PC2 explaining 5.32% of the variance. C) Screening of overlapping genes. D) Construction of the PPI network.

In the PCA, the pre-correction plot (Fig. 1A) revealed that PC1 and PC2 accounted for 96.78% and 2.13% of the variance, respectively. Samples from different datasets (GSE48452, GSE63067, and GSE66676) exhibited significant separation along the PC1 axis, indicating that batch effects substantially influenced inter-sample variation. The corrected PCA plot (Fig. 1B) demonstrates that PC1 and PC2 explained 15.3% and 5.32% of the variance. Following batch effect correction, the sample distribution became more uniform. Samples from the three datasets exhibited more integrated clustering on the PC1-PC2 plane, indicating the effective removal of batch effects.

For differential gene screening, 381 significantly differentially expressed genes were identified using thresholds of absolute logFC >0.5 and p-value <0.05. These differentially expressed genes showed significant expression variations across the three datasets and may be associated with relevant biological processes. Through batch effect correction, we ensured that the screened differentially expressed genes were not affected by inter-batch variations, thereby enhancing the reliability and stability of the results.

### Bisphenol A-MASLD target screening

After removing duplicates, 878 BPA targets were identified from the SwissTargetPrediction, PharmMapper, and ChEMBL databases. The OMIM database yielded 2,950 disease genes, whereas GeneCards provided 853 disease genes. After deduplication and merging of these two databases, 3,487 disease targets were obtained. The GEO dataset contributed to 381 disease targets. The intersection between disease targets from the GeneCards/OMIM and GEO datasets was defined as the total number of disease targets. The subsequent intersection with Bisphenol A targets yielded 34 overlapping genes (Fig. 1C).

### Construction of PPI network and identification of Core targets

**Fig. 2:**
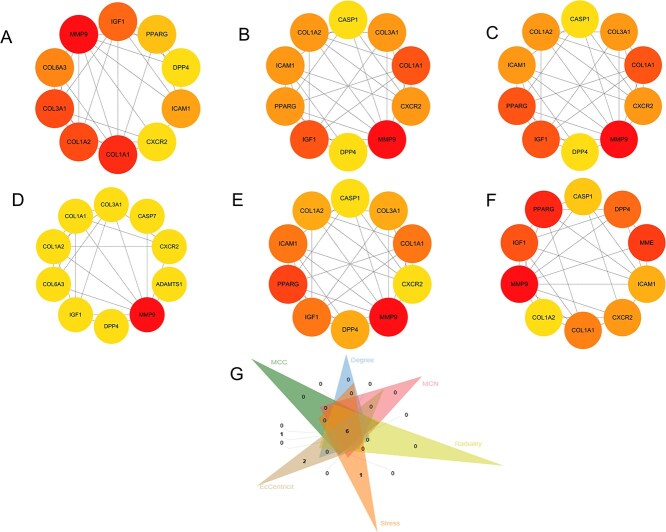
A–F) present the top 10 genes ranked using MCC, MNC, degree, Eccentricity, radiality, and stress algorithms. G) Key targets identified through the intersection analysis of targets obtained from the six algorithms.

To further analyze the key targets of MASLD, we constructed a PPI network for these 34 relevant targets (Fig. 1D). Using the Cytohubba plugin, we applied the MCC, MNC, Radiality, Stress, BottleNeck, and EcCentricity algorithms to identify the top 10 genes. By taking the intersection of the resulting genes, we screened six core targets: MMP9, COL1A1, COL1A2, IGF1, DPP4, and CXCR2 (Fig. 2).

### Machine learning analysis

**Fig. 3:**
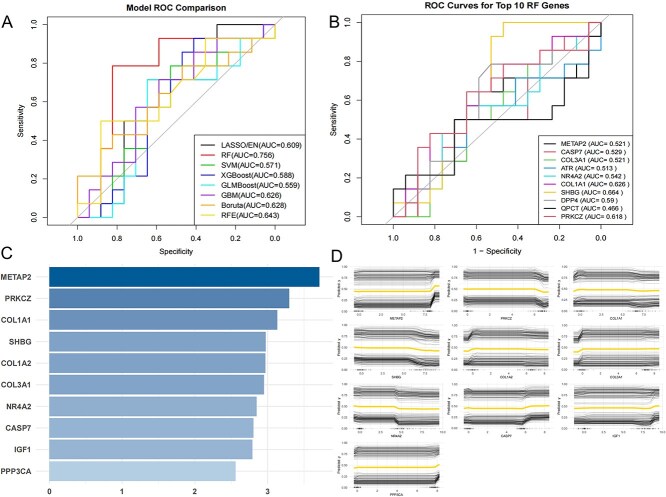
A) Comparison of ROC curves across various models, including LASSO/EN, RF, SVM, XGBoost, GLMBoost, GBM, Boruta, and RFE models, displaying their respective AUCs; B) ROC curves for the top 10 RF genes, presenting AUC values for METAP2, PRKCZ, COL1A1, and other genes; C) gene-specific ranking bar chart illustrating the specificity of the top 10 genes identified by the RF model; D) SHAP dependence plots depicting the relationship between SHAP values and feature values for the top 10 genes, analyzing each feature's contribution to model predictions.

We systematically evaluated the classification performance of multiple machine learning models and further investigated the predictive value of key genes within the Random Forest (RF) model. As shown in Fig. 3A, different models exhibited varying levels of performance, with the RF model achieving the best overall result (AUC = 0.756), reflecting superior predictive capacity and generalization performance. Figure 3B illustrates the single-gene ROC curves of the top ten important genes identified by the RF model. Although the individual AUC values of these ten genes were relatively modest, the RF model demonstrated strong overall stability and discriminative ability, suggesting that it remains highly reliable when integrating multiple features for combined prediction. Among them, SHBG (AUC = 0.664), COL1A1 (AUC = 0.626), and PRKCZ (AUC = 0.617) all exhibited AUC values above 0.6, indicating relatively strong classification capacity and practical predictive value. Further SHAP (Shapley Additive Explanation) feature importance analysis (Fig. 3C) revealed that these three genes ranked among the top features, highlighting their central roles in model predictions. The SHAP dependence plots (Fig. 3D) provided comprehensive information about the feature dependency of each gene, demonstrating clear positive correlations between gene expression levels and predicted probabilities—thereby enhancing the biological interpretability and rationality of the model. Ultimately, the three key genes identified through machine learning overlapped with one of the six core targets identified earlier—COL1A1. This finding is particularly significant, as it indicates that COL1A1 not only occupies a central position within the protein–protein interaction (PPI) network but also exhibits strong predictive power for disease status in the machine learning model. This cross-methodological convergence points to the critical function of COL1A1 in BPA-induced MASLD, reflecting its dual significance in both mechanistic regulation and clinical prediction.

**Fig. 4:**
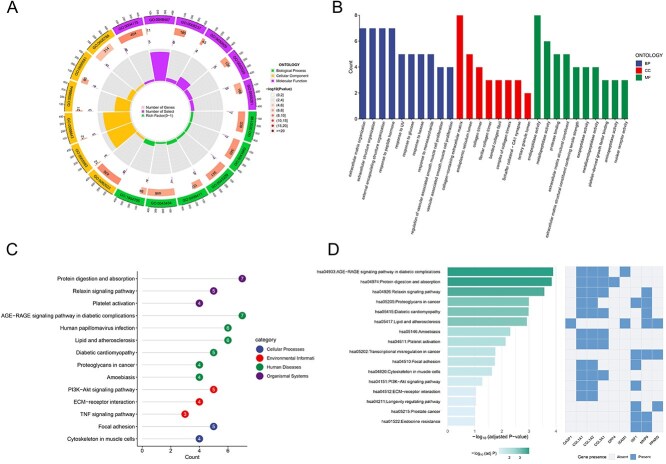
A-B) GO enrichment analysis results; C) pathway enrichment analysis of potential targets; D) pathway enrichment analysis of the top 10 potential targets by degree value.

**Fig. 5:**
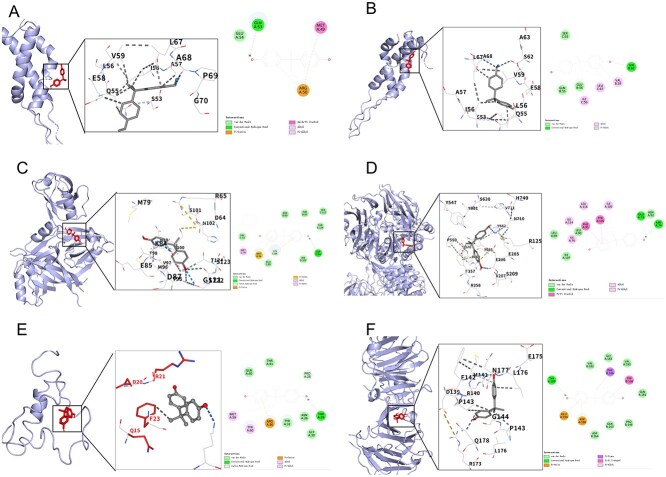
Molecular docking visualization: A) BPA-COL1A1; B) BPA-COL1A2; C) BPA-IGF1; D) BPA-MMP9; E) BPA-DPP4; F) BPA-CXCR2. Note: Standardized hyphen spacing and gene nomenclature.

### GO/KEGG enrichment analysis

The 34 overlapping genes were analyzed using ‘*H. sapiens*’ as the filtering criterion (Fig. 4A-B). GO functional enrichment analysis identified 197 biological processes (BP), 21 cellular components (CC), and 53 molecular functions (MF). The top 10 entries with the lowest p-values in each category were selected for visualization. BP terms primarily involve extracellular matrix and vascular structural remodeling, along with responses to metabolic signals and environmental stress. CC terms mainly focus on collagen-centered extracellular matrix structures. MF terms primarily encompassed proteolytic functions, extracellular matrix structural organization, and signaling factor-binding regulation. This reflects the potential of the sample for active matrix remodeling, signal transduction, and transcriptional response.

KEGG pathway enrichment analysis of the 34 potential targets revealed significantly enriched pathways (lowest *P-values*): the AGE-RAGE signaling pathway, PI3K-Akt signaling pathway, and pathways related to lipid metabolism and atherosclerosis, all of which are closely associated with metabolic steatotic liver disease (Fig. 4C). Particular attention should be paid to the PI3K-Akt signaling pathway, which is directly involved in insulin signal transduction, lipogenesis, glucose metabolism, and regulation of apoptosis and autophagy, constituting one of the core pathogenic mechanisms in MASLD.

### Pathway enrichment analysis of Core targets

Pathway enrichment analysis of the top 10 targets by degree value revealed that the relaxin signaling, protein digestion and absorption, and PI3K-Akt signaling pathways were closely associated with MASLD development (Fig. 4D). The PI3K-Akt signaling pathway again demonstrated critical importance, highlighting its mechanistic significance in BPA-induced MASLD. Within this pathway, COL1A1, COL1A2, and IGF1 played central roles, corresponding to the six core genes identified previously. COL1A1 was the predominant contributor. These findings provide valuable insights into the molecular mechanisms underlying BPA-induced MASLD, facilitating a deeper understanding of MASLD pathogenesis.

### Molecular docking results

In this study, six core targets—MMP9, COL1A1, COL1A2, IGF1, DPP4, and CXCR2—identified from the PPI network were prioritized for molecular docking analysis. The PPI network analysis aimed to identify key “hub” proteins occupying topological centers within the BPA–MASLD interaction network, as such targets are more likely to play central regulatory roles in complex pathological processes.

Molecular docking was performed using AutoDock Vina 1.2.2, through which binding sites and receptor-ligand interactions were determined. Binding energies lower than −5 kcal/moL were considered indicative of significant receptor–ligand affinity. The results showed binding energies of −6.4 kcal/moL for COL1A1, −5.7 kcal/moL for COL1A2, −5.7 kcal/moL for IGF1, −6.4 kcal/moL for MMP9, −7.3 kcal/moL for DPP4, and − 6.4 kcal/moL for CXCR2 (Fig. 5). These values demonstrate strong binding stability between Bisphenol A and all six core targets. The docking interactions were subsequently visualized to illustrate the spatial binding conformations and intermolecular interactions.

### Molecular dynamics simulation results

**Fig. 6:**
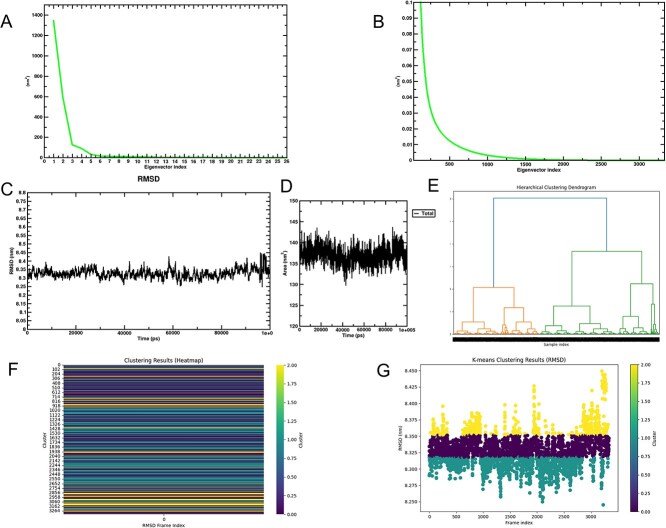
A) Variation curve of the first 26 principal components in principal component analysis (PCA); B) variation curve of principal components 27 to 3,333 in PCA; C) root mean square deviation (RMSD) analysis results; D) temporal changes in solvent-accessible surface area (SASA); E) hierarchical clustering dendrogram; F) RMSD-based heatmap; G) K-means clustering results.

Although the binding energy between DPP4 and BPA was slightly lower than that between COL1A1 and BPA in molecular docking, COL1A1 was selected for molecular dynamics (MD) simulation due to its higher centrality within the PPI network and superior predictive performance in the machine learning model (AUC > 0.6). This analysis aimed to further validate the binding stability of the COL1A1–BPA complex. Principal component analysis (PCA) of the simulated system showed that the first 26 principal components (PCs) captured the majority of the variance, indicating that these dominant directions govern the dynamic behavior of the system. The contribution of the subsequent PCs progressively declined (Fig. 6A and B), signifying their diminished importance. To present the data clearly, we visualized the first 26 components and the subsequent 3,327 components from the PCA analysis. The figure displays the variations in the first 26 PCA components and subsequent components with minor contributions.

RMSD analysis. The Root Mean Square Deviation (RMSD) was employed to evaluate the structural stability of the ligand-receptor system. During the 200 ns simulation period, the RMSD values stabilized between 8.30 and 8.40 nm (Fig. 6C), indicating system convergence without significant structural changes.

SASA analysis. Solvent-accessible surface area (SASA) analysis revealed relatively stable molecular surface area fluctuations over time. This stability further supports the robustness of the system during the 200 ns molecular dynamics (MD) simulation. The results indicated stable ligand-receptor interactions with no significant variation in the solvent effect (Fig. 6D).

Cluster analysis. Hierarchical clustering based on the RMSD analysis (Fig. 6E–G) further corroborated the stability of the simulation. The dendrogram from hierarchical clustering (Fig. 6E) revealed clustering relationships among samples from different time periods, demonstrating the maintenance of multiple similar conformations during the simulation. The heatmap (Fig. 6F) clearly displays the clustering results of samples across time frames, with color differentiation distinguishing cluster groups and indicating inter-sample similarities. The K-means clustering results (Fig. 6G) further indicate that the RMSD values of the system remained relatively stable throughout most of the simulation periods. Occasional cluster changes observed in a few frames suggest that the system underwent localized structural adjustments.

### Cellular infiltration analysis results

**Fig. 7:**
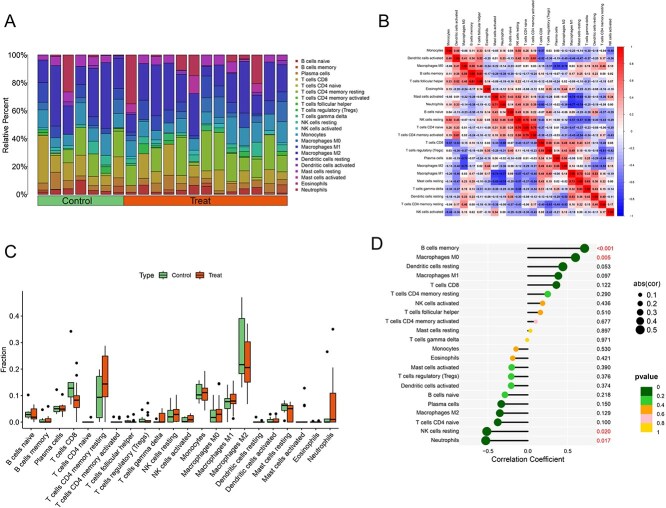
A) Composition of 22 immune cell subtypes across all samples; B) correlation heatmap illustrating the relationships among immune cell subtypes; C) boxplots showing the differences in infiltration levels of 22 immune cell types between the control and BPA-treated groups; D) correlation analysis between COL1A1 expression and the infiltration levels of 22 immune cell subtypes.

Immune cell infiltration analysis based on the CIBERSORT algorithm revealed distinct differences in immune cell composition between the control and BPA-treated groups. As shown in Fig. 7A  *T*  *cells CD4 memory resting*, *Macrophages M0*, *Macrophages M2*, and *B cells memory* were identified as the predominant infiltrating immune cell types. The correlation heatmap (Fig. 7B) demonstrated complex interrelationships among immune cells. A significant negative correlation was observed between M0 and M2 macrophages, suggesting that BPA exposure may induce a polarization shift between macrophage subtypes. Similarly, neutrophils exhibited a strong negative correlation with M2 macrophages, indicating that BPA might promote the upregulation of pro-inflammatory cells and the suppression of anti-inflammatory populations. In contrast, *activated CD4 memory T cells* showed a strong positive correlation with *naive CD4 T cells*, reflecting enhanced adaptive immune activation. Compared with healthy controls, the BPA-treated group exhibited increased proportions of M0 macrophages and neutrophils, alongside decreased proportions of M2 macrophages and resting CD4 memory T cells (Fig. 7C). To further explore the potential role of the core gene COL1A1 within the immune microenvironment, we analyzed its correlations with the infiltration levels of 22 immune cell types (Fig. 7D). The results showed that COL1A1 expression was significantly positively correlated with *memory B cells* and *M0 macrophages*, while displaying significant negative correlations with *neutrophils* and *resting NK cells*. Collectively, these findings suggest the presence of a complex immune regulatory network, in which the core gene COL1A1 may act as a pivotal mediator linking immune activation, tolerance, and chronic inflammation. Through modulating immune cell function and infiltration patterns, COL1A1 may play a bridging role in the immune response dynamics associated with BPA-induced MASLD.

## Discussion

Our study established a significant positive correlation between elevated BPA levels and an increased MASLD risk using NHANES data analysis. By integrating multiple databases and network analysis tools (including SwissTargetPrediction, PharmMapper, GEO, ChEMBL, and GeneCards), we screened 34 potential targets linking BPA to MASLD development. Further analysis through PPI network and machine learning identified key targets MMP9, COL1A1, COL1A2, IGF1, DPP4, and CXCR2 were identified as critically involved in the pathogenesis of MASLD, with COL1A1, COL1A2, and IGF1 identified as core targets.

KEGG pathway enrichment analysis revealed the significance of the PI3K/AKT signaling pathway. Cellular infiltration analysis indicated that Bisphenol A plays a crucial role in the function and polarization of immune cells. In this study, we observed significantly higher infiltration levels of M0 macrophages and neutrophils in the BPA-treated group compared with healthy controls. Previous in vitro studies have shown that BPA markedly upregulates the expression of pro-inflammatory cytokines such as TNF-α and IL-6 in macrophages, promoting their polarization toward the M1 phenotype, thereby enhancing the pro-inflammatory activation of previously unpolarized (M0-type) macrophages.^23^ Furthermore, BPA exposure has been reported to alter the expression of surface markers (e.g. CD11c, TLR4) on human neutrophils, suggesting that it modulates their immunophenotype and functional status to participate in inflammatory processes.^24^ Animal experiments further support this observation, indicating that BPA enhances neutrophil infiltration into tissues, thereby amplifying local inflammatory responses.^25^

Through KEGG pathway enrichment analysis, we identified the PI3K/AKT signaling pathway as a key mechanism underlying BPA-induced immune alterations. Studies indicate that hepatic macrophages (including resident Kupffer cells and peripheral monocyte-derived macrophages) play a central role in the immune microenvironment of MASLD.^26^ The PI3K/AKT signaling pathway promotes M2 polarization of macrophages. Cytokines secreted by these polarized macrophages, such as IL-10 and TGF-β, contribute to fibrosis progression by activating hepatic stellate cells (HSCs) and inducing collagen deposition.^27^ PI3K/AKT pathway regulates lipid metabolism and balances nutritional stress in hepatocytes, indirectly influencing immune cell function. Lipid overload and hyperglycemic environments activate PI3K/AKT, inducing endoplasmic reticulum and inflammatory stress responses. This triggers the release of DAMPs and chemokines, recruiting immune cells to the liver and activating Kupffer cells and peripheral macrophages. Concurrently, regulatory molecules such as NUP85 mediate the upregulation of CCR2 expression through the PI3K/AKT signaling pathway, enhancing inflammatory cell migration and further promoting the progression of MASLD.^28^ The regulation of adaptive immunity by the PI3K/AKT/mTOR signaling pathway is equally critical. Its activation promotes the differentiation of CD4^+^ T cells into the pro-inflammatory Th17 subtype and upregulates IL-17 production, thereby contributing to the occurrence of liver injury and fibrosis in NASH.^29^ Furthermore, the regulation of autophagy by the PI3K/AKT signaling pathway is associated with the progression of MASLD. Autophagy is a cellular self-protective mechanism that clears damaged organelles and excess lipids. In MASLD, aberrant activation of PI3K/AKT promotes sustained lipid accumulation and cellular damage by inducing mitophagy.^30^ Furthermore, studies have demonstrated that the PI3K/AKT signaling pathway plays a significant role in the initiation and progression of liver fibrosis. This pathway promotes the activation and proliferation of hepatic stellate cells (HSCs), leading to excessive deposition of extracellular matrix (ECM) components, thereby exacerbating the process of hepatic fibrosis.^31^ Given that COL1A1, COL1A2, and IGF1 are all involved in the PI3K/AKT signaling pathway, these genes are likely to play crucial roles in the complex mechanisms of BPA-induced MASLD, with COL1A1 occupying a particularly prominent position. Notably, a partial discrepancy was observed between the core targets identified by the PPI network analysis and those obtained through machine learning, reflecting the complementary and distinctive perspectives of the two methodologies in target identification. The PPI network analysis focuses on protein–protein interaction topology, emphasizing the structural centrality of targets within the pathological network, making it particularly suitable for uncovering mechanistic core nodes. In contrast, the machine learning approach emphasizes gene expression features associated with disease classification, thereby highlighting their potential utility in clinical prediction and molecular subtyping. As a shared target identified by both analytical strategies, COL1A1 demonstrates dual significance—serving as both a mechanistic regulator within the molecular network and a predictive biomarker with translational potential.

The COL1A1 gene encodes the α1 chain of type I collagen, which is the primary component of extracellular matrix (ECM) accumulation in fibrosis. Its expression is markedly elevated during MASLD progression and is positively correlated with hepatic stellate cell (HSC) activation and fibrosis severity. The excessive synthesis of COL1A1 constitutes a critical driving factor in the development of hepatic fibrosis and liver cirrhosis.^32^^,^^33^ In MASLD, COL1A1 expression is regulated by multiple molecular signaling pathways. Specifically, the TGF-β1 (Transforming Growth Factor Beta 1) signaling pathway promotes transcriptional upregulation and synthesis of COL1A1 through activation of Smad-dependent pathways.^34^ Furthermore, the immune-metabolic circuit formed by activated macrophages and T cells secretes growth factors such as TGFβ and PDGF. These factors act on hepatic stellate cells (HSCs) and activate the PI3K/AKT signaling pathway This process enhances protein expression of COL1A1 and COL1A2, increases extracellular matrix (ECM) synthesis, and ultimately exacerbates fibrosis.^35^^,^^36^ The immune infiltration analysis revealed a significant positive correlation between COL1A1 expression and memory B-cell infiltration, suggesting that COL1A1 may exert broader immunomodulatory effects through its influence on memory B-cell function. Previous studies have reported that B cells can promote fibroblast-derived COL1A1 production by secreting cytokines such as BAFF (B-cell activating factor), indicating that B cells may positively regulate extracellular matrix (ECM) synthesis through immune–stromal crosstalk.^37^ In chronic inflammatory or fibrotic environments, type I collagen structures enriched in COL1A1 can activate the NF-κB signaling pathway via integrin receptors (e.g. α2β1), thereby modulating immune cell functions.^38^ Moreover, growing evidence indicates that the NF-κB pathway plays a central regulatory role in B-cell development, activation, and memory formation.^39^ Taken together, these findings suggest that COL1A1 may participate in B cell–mediated immune regulation within the chronic inflammatory microenvironment of MASLD through an ECM–integrin–NF-κB signaling network, bridging extracellular matrix remodeling with immune activation processes. COL1A2, a core component of the extracellular matrix (ECM), is synthesized in large quantities along with COL1A1 during hepatic stellate cell (HSC) activation. Upon activation, HSCs transform into a fibroblastic phenotype, initiating massive synthesis of COL1A2 which deposits into the ECM to form nodular structures characteristic of hepatic fibrosis.^40^ Therefore, COL1A1 and COL1A2 are recognized as key molecular markers for MASLD fibrosis, and their expression levels can be used to assess hepatic fibrosis severity and predict therapeutic responses.^41^^,^^42^

Insulin-like growth factor 1 (IGF1) is a protein primarily synthesized by the liver that belongs to the growth factor family. According to a systematic review and meta-analysis, serum IGF1 levels in MASLD patients were significantly lower than those in the healthy control group, with this difference being particularly pronounced in non-alcoholic steatohepatitis (NASH) patients^43^. IGF1 activates the PI3K/AKT signaling pathway via its receptor IGF1R, thereby regulating cell proliferation, survival, and metabolism. During hepatic fibrogenesis, IGF1 promotes the activation of hepatic stellate cells (HSCs) and their transformation into a fibroblastic phenotype through this pathway, driving liver fibrosis progression.^44^ Molecular docking confirmed stable binding between Bisphenol A and COL1A1, COL1A2, and IGF1, while molecular dynamics simulations highlighted the particularly favorable binding affinity between COL1A1 and Bisphenol A. This suggests that Bisphenol A may directly interact with the protein products of these three genes to induce the onset and progression of MASLD, with COL1A1 playing a critical role.

Although this study revealed the potential mechanisms of BPA-induced MASLD and provided an in-depth exploration of the crucial roles of the PI3K/AKT signaling pathway, COL1A1, COL1A2, and IGF1 in hepatic fibrosis, several limitations remain. First, the research primarily relied on database and network analysis tools to screen potential targets, lacking comprehensive experimental validation. Future studies could further verify the roles of these targets using animal models and clinical samples. Second, although this study explored the mechanisms of immune response, the specific functions and interactions of immune cell types remain insufficiently elucidated. This knowledge gap critically impedes our understanding of the immune microenvironment of MASLD.

## Conclusions

This study provides profound insights into the complex mechanisms underlying BPA-induced MASLD, revealing the pivotal role of the PI3K/AKT signaling pathway. This further highlights the critical involvement of COL1A1, COL1A2, and IGF1 in this pathological process, with COL1A1 playing a particularly critical role. Cellular infiltration analysis further indicated that immune responses significantly contribute to the pathogenesis of MASLD. BPA modulates immune cell polarization and alters the immune microenvironment via the PI3K/AKT pathway, thereby accelerating hepatic fibrosis progression. The interplay between these immune responses and the PI3K/AKT signaling pathway provides novel therapeutic targets and research directions for elucidating the mechanisms underlying MASLD.

### Institutional review board statement

Ethical review and approval were waived for this study due to the exclusive use of publicly available, de-identified datasets. The study did not involve any direct interaction with human participants or the use of personally identifiable information. All data were obtained from open-access databases, which have their own ethical approvals and data access regulations.

### Informed consent statement

All data used in this study were obtained from publicly available and anonymized databases, including NHANES, PubChem, GeneCards, GEO, and other open-access databases, which do not contain personally identifiable information and therefore do not require informed consent.

## Data Availability

The data are contained within the article and Supplementary Materials.
